# Human Case of *Ehrlichia chaffeensis* Infection, Taiwan

**DOI:** 10.3201/eid2511.190665

**Published:** 2019-11

**Authors:** Shih-Huan Peng, Su-Lin Yang, Yu-Ni Ho, Hsiang-Fei Chen, Pei-Yun Shu

**Affiliations:** Centers for Disease Control, Taipei, Taiwan (S.-H. Peng, S.-L. Yang, H.-F. Chen, P.-Y. Shu);; Chang Gung Memorial Hospital–Kaohsiung Medical Center, Kaohsiung, Taiwan (Y.-N. Ho)

**Keywords:** Ehrlichia chaffeensis, human monocytic ehrlichiosis, vector-borne infections, tickborne diseases, Taiwan, bacteria, rickettsia

## Abstract

In 2018, an immunosuppressed woman in southern Taiwan had onset of fever, chills, myalgia, malaise, thrombocytopenia, lymphocytopenia, and elevated hepatic transaminases. Investigation revealed infection with *Ehrlichia chaffeensis*. This autochthonous case of human monocytotropic ehrlichiosis was confirmed by PCR, DNA sequencing, and seroconversion.

Human monocytic ehrlichiosis (HME) is an acute, febrile, tickborne disease caused by the bacterium *Ehrlichia chaffeensis*. HME was first reported in the United States in 1986 (*1*), and >1,000 ehrlichiosis cases have been reported annually since 2012 (https://www.cdc.gov/ehrlichiosis/stats/index.html). In Asia, however, only a limited number of HME cases have been reported in 3 countries (Thailand, South Korea, and China) (*2*–*4*). 

Clinical manifestations of HME range from mild febrile illness to severe multiple organ failure. The most common symptoms of HME are fever, headache, myalgia, malaise, nausea, vomiting, diarrhea, and abdominal pain (*5*–*7*), which are difficult to differentiate from the symptoms of other febrile infectious diseases. Therefore, HME must be confirmed by laboratory diagnosis. 

Although HME has not been documented in Taiwan, serologic evidence of *Ehrlichia* spp. has been detected in small mammals, such as *Rattus norvegicus*, *R. losea*, and *Bandicota indica* rats that are found around international and local harbors (*8*). In addition, *Haemaphysalis flava* ticks infected with *Ehrlichia* spp. have been collected from pale thrush birds (*Turdus pallidus*) and identified in Taiwan (*9*). We report an autochthonous human case of *E. chaffeensis* infection in Taiwan.

In mid-July 2018, a 66-year-old woman living in the Namaxia District of Kaohsiung City in southern Taiwan was admitted to Kaohsiung Chang Gung Memorial Hospital with a 5-day history of intermittent fever (39.8°C), chills, myalgias, malaise, mild dyspnea, and diffuse abdominal pain. The patient had underlying hypertension, type 2 diabetes mellitus, alcoholic fatty liver, and gastroesophageal reflux disease. Laboratory examinations at admission showed that the patient had thrombocytopenia; lymphocytopenia; elevated levels of C-reactive protein, aspartate aminotransferase, alanine aminotransferase, and creatinine; and an increased number of polymorphonuclear leukocytes (Table). Whole blood counts were within reference ranges, and no leukocytopenia was observed. A chest radiograph showed mild infiltration over the bilateral lower lung fields. Laboratory tests for dengue, influenza A and B, hepatitis A, hepatitis B, and hepatitis C viruses were all negative. She was admitted under the impression of atypical infection and thrombocytopenia.

**Table:** Laboratory and diagnostic findings for a human case of *Ehrlichia chaffeensis* infection, Taiwan*

Laboratory or diagnostic finding	Patient value or result	Reference value or method
Leukocytes	5,800/μL	3.9–10.6 × 10^3^/μL
Red blood cell	4,950,000/μL	3.9–5.4 × 10^6^/μL
Hemoglobin	14.3 g/dL	12–16 g/dL
Platelets	27,000/μL	150–400 × 10^3^/μL
Segment	82.7%	42%–74%
Lymphocyte	13%	25%–56%
Creatinine	1.16 mg/dL	0.44–1.03 mg/dL
Aspartate aminotransferase	97 U/L	0–37 U/L
Alanine aminotransferase	71 U/L	0–40 U/L
C-reactive protein	131.2 mg/L	<5 mg/L
Dengue virus	Negative	Rapid test, ELISA, PCR
Influenza virus	Negative	Antigen
HAV/HBV/HCV	Negative	HAV IgM (ECLIA), HBV HBsAg (ECLIA), HCV Anti-HCV (ECLIA)
*Leptospira interrogans*	Negative	MAT or isolation
*Coxiella burnetii*	Negative	PCR or IFA
*Orientia tsutsugamushi*	Negative	PCR or IFA
*Rickettsia typhi/R. prowazekii*	Negative	PCR or IFA
*R. rickettsii*	Negative	IFA
*R. conorii*	Negative	IFA
*Anaplasma phagocytophilum*	Negative	PCR or IFA
*Ehrlichia chaffeensis*	Positive	PCR or IFA

*Diagnostic methods based on guidelines on standard operating procedure for laboratory diagnosis provided by Taiwan Centers for Disease Control (https://www.cdc.gov.tw). ECLIA: electrochemiluminescence immunoassay; HAV, hepatitis A virus, HBsAG, hepatitis B surface antigen; HBV, hepatitis B virus, HCV, hepatitis C virus; IFA, immunofluorescence assay; MAT, microscopic agglutination test.

The patient is a coffee farmer who lives in a rural region in Kaohsiung. Although she claimed not to have received arthropod or animal bites, small mammals and birds had often been seen around her workplace and house. Therefore, arthropodborne rickettsial diseases were suspected, and oral doxycycline (100 mg every 12 h) for 4 days and intravenous ceftriaxone (1 g every 12 h) for 7 days were prescribed as empirical therapy on the patient’s first day at the hospital. Because ehrlichial infection had not been confirmed during hospitalization, the patient was discharged with a prescription (500 mg cefadroxil monohydrate every 12 h) to be taken for 5 days because of suspicion of atypical bacterial infection.

Blood specimens collected from the patient on day 6 (acute-phase specimens) and day 20 (convalescent-phase specimens) after illness onset were sent to the Taiwan Centers for Disease Control (Taipei, Taiwan) for laboratory diagnosis of zoonotic diseases. DNA extracted from acute-phase blood specimens using the QIAamp DNA blood Mini Kit (QIAGEN GmbH, https://www.qiagen.com) was used to detect *Ehrlichia chaffeensis* infection using a primer set targeting ehrlichial 16S rRNA gene (forward primer: AGCGGCTATCTGGTTCGA; reverse primer: CATGCTCCACCGCTTGTG) and an *E. chaffeensis*–specific primer set targeting the nitrogen assimilation regulatory protein (*ntrX*) gene (forward primer: TGCCGGTAGATATAGTATCGA; reverse primer: ATTTGCGATGAAGTGCGG) by QuantiNova SYBR green real-time PCR (QIAGEN). The PCR products of 16S rRNA (182 bp; GenBank accession no. MN088851) and the *ntrX* gene sequence (153 bp; GenBank accession no. MN096569) were determined and analyzed. The sequence was 100% homologous with the sequences of *E. chaffeensis* strains, including the Arkansas, Jax, Saint Vincent, West Paces, Wakulla, Osceola, Liberty, and Heartland strains. The PCR results were negative for *Coxiella burnetii*, *Orientia tsutsugamushi*, typhus group rickettsiae, spotted fever group rickettsiae, and *Anaplasma phagocytophilum* (Appendix Table). Paired (acute- and convalescent-phase) serum samples were used to detect antibodies against *E. chaffeensis* by using indirect immunofluorescence assay according to the manufacturer’s recommendation (Focus Diagnostics, https://www.focusdx.com). IgG against *E. chaffeensis* showed seroconversion (titers ranging from <1:16 to 1:256) of the paired serum samples. IgG against *Coxiella burnetii, Orientia tsutsugamushi*, typhus group rickettsiae, spotted fever group rickettsiae, and *Anaplasma phagocytophilum* were all negative. The results of the microscopic agglutination test and the isolation of *Leptospira *sp*.* were also negative.

The presence of an HME case highlights the need for further studies of the prevalence, geographic distribution, and control of this disease in Taiwan. Human monocytic ehrlichiosis patients with immunosuppressive conditions, such as diabetes, might have a higher risk for hospitalization and life-threatening complications (*10*). In this case, the suspicion of rickettsial infection was based on the patient’s potential exposure to arthropodborne pathogens at her workplace and home, and the patient responded quickly to doxycycline treatment. Physician awareness of HME and early diagnosis and treatment are essential to improve disease outcomes. 

Appendix Additional information on a human case of *Ehrlichia chaffeensis* infection, Taiwan.
