# Supplementary material for: Human Case of Ehrlichia chaffeensis Infection, Taiwan
Source: Emerg Infect Dis. 2019 Nov;25(11):2141–3. doi: 10.3201/eid2511.190665 (PMC6810210; doi:10.3201/eid2511.190665)
Supplement: Appendix — Additional information on a human case of Ehrlichia chaffeensis infection, Taiwan. [file 19-0665-Techapp-s1.pdf]

Article DOI: https://doi.org/10.3201/eid2511.190665

Human Case of *Ehrlichia chaffeensis* Infection, Taiwan

Appendix

Appendix Table. Primers used for SYBR green real-time PCR assay in this study

Primer	Target gene (organism)	Sequence (5′ to 3′)	Amplicon (bp)	Reference
DN-F	Capsid(C) (dengue virus)	CAATATGCTGAAACGCGAGAGAAA	171	(*1*)
DN-R		CCCCATCTATTCAGAATCCCTGCT		
EC-F1	16S rRNA (*Ehrlichia* spp.)	AGCGGCTATCTGGTTCGA	218	This study*
EC-R1		CATGCTCCACCGCTTGTG		
EC-F2	ntrX (*Ehrlichia chaffeensis*)	TGCCGGTAGATATAGTATCGA	192	This study*
EC-R2		ATTTGCGATGAAGTGCGG		
Trans1	IS1111 (*Coxiella burnetii*)	TATGTATCCACCGTAGCCAGTC	687	(*2*)
Trans2		CCCAACAACACCTCCTTATTC		
261F	IS1111 (*C. burnetii*)	GAGCGAACCATTGGTATCG	203	(*2*)
463R		CTTTAACAGCGCTTGAACGT		
OTF7	16S rRNA (*Orientia tsutsugamushi*	CCAGYGGGTRATGCCGGGAACTAT	276	(*3*)
OTR6	and *Rickettsia spp.*)	GGCAGTGTGTACAAGGCCCGAGAA		
RST-14F	TSA56 (*O. tsutsugamushi*)	CCATTTGGTGGTACATTAGCTGCAGGT	233	(*4*)
RST-6R		TCACGATCAGCTATACTTATAGGCA		
RT-F	17kDa (*R. typhi*)	GGGTGGTATGAACAAACAAGGGACTG	240	†
RT-R		CGCCATTCTATGTTACTACCGCTAGG		
RP-F	17kDa (*R. prowazekii*)	TGGTCAGAGTGGTATGAACAAACAAG	246	†
RT-R		CGCCATTCTATGTTA CTACCGCTAGG		
SFG-F	17kDa (Spotted fever group)	GGTATGAATAAACAAGGTACAGGAAC	306	†
SFG-R		ATATTGACCAGTGCTATTTCTATAAG		

Primer	Target gene (organism)	Sequence (5′ to 3′)	Amplicon (bp)	Reference
AP-F1	msp2 (*Anaplasma phagocytophilum*)	ACGTTAGCGCTTTGGAGACT	300	†
AP-R1		TCTTGAAGCGCTCGTAACCA		
903f	msp2 (*Anaplasma phagocytophilum*)	AGTTTGACTGGAACACACCTGATC	122	(*5*)
1024r		CTCGTAACCAATCTCAAGCTCAAC		

*The *E. chaffeensis* Arkansas strain was served as positive control for PCR assay. DNA was extracted from acute-phase blood specimens using the QIAamp DNA blood Mini Kit (QIAGEN GmbH, Holden, Germany) according to the manufacturer's instructions. Real-time PCR amplification was performed using QuantiNova SYBR green real-time PCR kit (QIAGEN) with the following parameters: 95°C for 2 minutes (pre-incubation), 40 cycles of 95°C for 5 seconds (denaturation), 60°C for 10 sec (annealing and extension) and melting curve analysis (95°C for 1 minute, lowered to 68°C for 30 seconds and followed by a gradual increase in temperature to 95°C with continuous recording of fluorescence). The results were analyzed with the software program of the LightCycler 96 Real-Time PCR system (Roche Diagnostics, Mannheim Germany).

†Diagnostic methods based on guidelines on standard operating procedure for laboratory diagnosis provided by Taiwan Centers for Disease Control (https://www.cdc.gov.tw).

References

1. Shu PY, Chang SF, Kuo YC, Yueh YY, Chien LJ, Sue CL, et al. Development of group- and serotype-specific one-step SYBR green I-based real-time reverse transcription-PCR assay for dengue virus. J Clin Microbiol. 2003;41:2408–16. PubMed https://doi.org/10.1128/JCM.41.6.2408-2416.2003

2. Parisi A, Fraccalvieri R, Cafiero M, Miccolupo A, Padalino I, Montagna C, et al. Diagnosis of *Coxiella burnetii*-related abortion in Italian domestic ruminants using single-tube nested PCR. Vet Microbiol. 2006;118:101–6. PubMed https://doi.org/10.1016/j.vetmic.2006.06.023

3. Yang SL, Tsai KH, Chen HF, Luo JY, Shu PY. Evaluation of enzyme-linked immunosorbent assay using recombinant 56k-Da type-specific antigens derived from multiple *Orientia tsutsugamushi* strains for detection of scrub typhus infection. Am J Trop Med Hyg. 2019;100:532–9. PubMed https://doi.org/10.4269/ajtmh.18-0391

4. Tsai KH, Lu HY, Tsai JJ, Yu SK, Huang JH, Shu PY. Human case of *Rickettsia felis* infection, Taiwan. Emerg Infect Dis. 2008;14:1970–2. PubMed https://doi.org/10.3201/eid1412.080515

5. Vargas-Hernandez G, André MR, Cendales DM, Sousa KC, Gonçalves LR, Rondelli MC, et al. Molecular detection of *Anaplasma* species in dogs in Colombia. Rev Bras Parasitol Vet. 2016;25:459–64. PubMed https://doi.org/10.1590/s1984-29612016066
